# Effects of Gestational Hypoxia on PGC1α and Mitochondrial Acetylation in Fetal Guinea Pig Hearts

**DOI:** 10.1007/s43032-023-01245-5

**Published:** 2023-05-03

**Authors:** Hong Song, Loren P. Thompson

**Affiliations:** Department of Obstetrics, Gynecology and Reproductive Sciences, University of Maryland, Baltimore, School of Medicine, 655 W. Baltimore St., Baltimore, MD 21201 USA

**Keywords:** Hypoxia, Fetal heart, Sexual dimorphism, Mitochondria, Fetal hypoxia

## Abstract

Chronic intrauterine hypoxia is a significant pregnancy complication impacting fetal heart growth, metabolism, and mitochondrial function, contributing to cardiovascular programming of the offspring. PGC1α (peroxisome proliferator-activated receptor γ co-activator 1α) is the master regulator of mitochondrial biogenesis. We investigated the effects of hypoxia on PGC1α expression following exposure at different gestational ages. Time-mated pregnant guinea pigs were exposed to normoxia (NMX, 21% O_2_) or hypoxia (HPX, 10.5% O_2_) at either 25-day (early-onset) or 50-day (late-onset) gestation, and all fetuses were extracted at term (term = ~65-day gestation). Expression of nuclear PGC1α, sirtuin 1 (SIRT1), AMP-activated protein kinase (AMPK), and mitochondrial sirtuin 3 (SIRT3) was measured, along with SIRT3 activity and mitochondrial acetylation of heart ventricles of male and female fetuses. Early-onset hypoxia increased (*P*<0.05) fetal cardiac nuclear PGC1α and had no effect on mitochondrial acetylation of either growth-restricted males or females. Late-onset hypoxia had either no effect or decreased (*P*<0.05) PCC1α expression in males and females, respectively, but increased (*P*<0.05) mitochondrial acetylation in both sexes. Hypoxia had variable effects on expression of SIRT1, AMPK, SIRT3, and SIRT3 activity depending on the sex. The capacity of the fetal heart to respond to hypoxia differs depending on the gestational age of exposure and sex of the fetus. Further, the effects of late-onset hypoxia on fetal heart function impose a greater risk to male than female fetuses, which has implications toward cardiovascular programming effects of the offspring.

## Introduction

Chronic intrauterine hypoxia is one of the most significant complications of pregnancy, altering normal placenta and fetal development and leading to fetal growth restriction [1, 2]. Tissue hypoxia disrupts cellular energy metabolism and mitochondrial dysfunction, both of which are at the core of generating oxidative stress and fetal organ dysfunction. Chronic hypoxia generates oxidative stress via electron leak along the respiratory chain, thereby reducing the efficiency of oxidative phosphorylation and altering mitochondrial function [3, 4].

The mitochondria are important cellular organelles for generating energy supply. In early gestation, the fetal heart is reliant on anaerobic glycolysis for ATP [5]. With maturation, the fetal heart increases its capacity for oxidative phosphorylation as it increases its mitochondrial density, ultrastructural organization [6], and cellular compartmentation of metabolic processes [5, 7, 8]. This assures an efficient energy transfer to contractile proteins in accordance with an increasing metabolic demand at the time of birth [5]. Chronic hypoxia disrupts mechanisms associated with both contractile [9, 10] and mitochondrial function in the fetal heart [11–14]. This has long-term consequences associated with cardiovascular programming of the offspring [15–18]. Thus, understanding how hypoxia alters mitochondrial function of the fetal heart is important for identifying the underlying mechanisms that initiate developmental programming of cardiovascular disease.

Peroxisome proliferator-activated receptor γ co-activator 1α (PGC1α) is the master regulator of mitochondrial biogenesis via regulation of both nuclear- and mitochondrial-encoded protein expression in several tissues including the heart [19–21]. Translocation of PGC1α into the nucleus is regulated by deacetylation by sirtuin 1 (SIRT1) and phosphorylation by AMP-activated protein kinase (AMPK) in the cytosol. While AMPK is a sensor of metabolic conditions, effects of both AMPK and SIRT1 on PGC1α transcription are mediated within the nucleus [19, 22]. Activated-nuclear PGC1α induces expression of transcription factors, such as NRF 1/2 and mitochondrial TFAM, which are transported to the mitochondrial DNA and regulate respiratory complex subunit and SIRT3 expression [19]. SIRT3 is the most important mitochondrial deacetylase, which regulates protein acetylation by acetyl Co A of respiratory complex subunits [23]. The link between PGC1α and nuclear-encoded SIRT3 is important in the regulation of mitochondrial protein acetylation and function [19].

We have previously reported that chronic intrauterine hypoxia disrupts the expression of mitochondrial respiratory complex subunits (CI, CIII, and CV) and alters CI and CIV activities in both left cardiac ventricles and isolated cardiomyocytes of fetal guinea pig hearts [11, 13]. Further, there is a sexual dimorphism in mitochondrial responses of fetal hearts to intrauterine hypoxia, depending on the gestational age of exposure (early vs late gestation) [13]. For example, exposure to hypoxia in early gestation (40–50 days, 65 days = term) increases the expression of mitochondrial CI, CIII, and CV subunits in male fetal heart ventricles, whereas exposure in late gestation decreases the expression of the same subunits in both male and female hearts [13]. These studies show that gestational timing of hypoxia can differentially affect mitochondrial protein expression and, potentially, impact cardiac mitochondrial function.

In adult heart, the importance of PGC1α in the regulation of mitochondrial biogenesis is well established [19–21]. Yet, its role in the fetal heart and the effects of gestational hypoxia on PGC1α expression has not been investigated. Given the differential effects of hypoxia on mitochondrial respiratory complex subunit expression shown in our previous study [13], our aim in this study was to determine the effects of hypoxia on upstream signaling of PGC1α as a mechanism of downstream mitochondrial protein activity in fetal hearts. We hypothesized that the hypoxia-induced increase in respiratory complex I, III, and V subunit expression would be mediated by increased PGC1α expression in early-onset hypoxia and decreased with late-onset hypoxia. We further aimed to determine sex differences in this signaling response to assess the fetuses’ vulnerability to the hypoxic challenges.

## Methods

All animal procedures using guinea pigs were approved by the University of Maryland Institutional Animal Care and Use Committee in accordance with AAALAC International accreditation (Animal Welfare Assurance no. A3200-01).

### Animal Model

Timed-mated pregnant guinea pigs were housed under conditions of either normoxia (room air, NMX) or chronic hypoxia (10.5% O_2_, HPX) in a normobaric plexiglass chamber, generating maternal [11] and fetal hypoxia, the latter evidenced by fetal cardiac HIF (hypoxia-inducible factor) signaling [24] and oxidative stress [25]. Oxygen levels were reduced in the chamber with N_2_ gas mixed with room air, monitored with an O_2_ sensing probe (Reming Bioinstruments, Redfield, NY) and a servo-controlled feedback regulator to maintain a stable 10.5% O_2_ level. Excess CO_2_ and H_2_O vapor were removed by exposing the air mixture to soda lime and silica gel, respectively, Animals were kept in individual box containers within the chamber so food and water, provided ad libitum, could be monitored and replenished every other day. All animals are housed in a temperature-controlled room with a light/dark cycle every 12 h. Similar to our previous study [13], animals were exposed to hypoxia at either 25-day or 50-day gestation (term = ~65-day gestation), corresponding to a duration of 39 and 14d, respectively. Exposure at 25-day gestation corresponds to early stages of trophoblast invasion (~21-day gestation and placental development) in the guinea pig, and exposure at 50-day gestation corresponds to the rapid fetal growth phase, post organogenesis, and placenta maturation [26]. At near term, pregnant guinea pigs were anesthetized (ketamine, 80mg/kg, s.c.; xylazine, 6mg/kg, s.c.) and fetuses extracted via an abdominal incision and uterotomy following subcutaneous administration of 2% lidocaine. Anesthetized fetuses were weighed and sexed. Fetal hearts were weighed and left ventricles excised and flash frozen in liquid N_2_ and stored in −80 ^ο^C freezer.

### Extraction of Nuclear and Mitochondrial Fraction from Fetal Heart Ventricles

Frozen fetal cardiac ventricles were used to obtain nuclear and mitochondrial protein fractions for western immunoblot analysis. Briefly, 20–30 mg frozen ventricle pieces were ground with a mortar pestle in liquid N_2_ and resuspended in 800 μl of ice-cold homogenization buffer. The nuclear fraction was obtained by using a modified method with two-time homogenization and centrifugation [27–29]. The pellet containing the nuclear fraction was resuspended in 1x RIPA Lysis Buffer (Millipore, Calbiochem, MA, USA) and supplemented with 1x protease and phosphatase inhibitor cocktail tablet (Roche Diagnostics, Munich, Germany). The mitochondrial fraction was isolated by a standard differential centrifugation method [11, 13]. The pellet containing the mitochondrial fraction was resuspended in 1x RIPA Lysis Buffer (Millipore) and supplemented with 1x protease inhibitor cocktail tablet (Roche). All sample protein concentrations were measured by using the Bio-Rad Protein Assay (Bio-Rad). These procedures generate nuclear and mitochondrial fractions [30] without contamination.

### Western Immunoblot

Protein expression was measured for nuclear fractions of PGC1α, SIRT1, phosphorylated-AMPK, total AMPK, and mitochondrial fractions of SIRT3. 20–30μg of total protein from either nuclear or mitochondrial fractions were loaded onto precast 4–12% Bis-Tris mini gels (Invitrogen, Waltham, MA) for gel electrophoresis and then transferred to a PVDF membrane. Nuclear proteins were detected by anti-PGC1α (1:1000), anti-SIRT1 (1:1000, Proteintech, Rosemont, IL), anti-AMPKa (1:1000), and anti-phospho-AMPK (Thr172) (1:1000, Cell Signaling Technology, Danvers, MA). Density values of each of the bands were normalized to their corresponding loading control, Lamin A/C (4C11) (1:2000, Cell Signaling Technology), and expressed as relative expression. Mitochondrial proteins were detected by anti-SIRT3 (1:1000, Abcam, Cambridge, MA) and anti-acetylated-lysine (Ac-K^2^-100) (1:1000, Cell Signaling Technology). Each of the bands was normalized to VDAC1/Porin (1:1000, Abcam) as a loading control and expressed as relative expression. A peroxidase-conjugated secondary antibody (SeraCare Life Sciences, Gaithersburg, MD) was used for all immunoblots.

Immunoprecipitation was performed to confirm respiratory complex I subunit was the acetylated mitochondrial protein at MW 20kDa. Mitochondrial protein was isolated from fetal heart left ventricle, and immunoprecipitation was performed using Dynabeads Protein A Immunoprecipitation Kit (Invitrogen, Waltham, MA) following the manufacturer’s protocol. Briefly, anti-acetylated-lysine (Ack) antibody at 1:100 dilution (Cell Signaling Technology) was mixed with 50 μl of Dynabeads and then incubated with 100 μl of protein lysate overnight. Lysates containing acetylated mitochondrial protein were probed with anti-NDUFB8 antibody (anti-complex I subunit, Abcam) at 1:20 dilution.

### Sirtuin Activity Assay

SIRT3 enzyme activity of mitochondrial fractions was measured using the SIRT3 Activity Assay Kit (Fluorometric, Abcam, Cambridge, MA) per the manufacturer’s protocol. Briefly, 50μg of mitochondrial protein was added into individual microplate wells containing a designated amount of fluorescence-labeled acetylated substrate peptide, nicotinamide adenine dinucleotide (NAD), and the developer. Fluorescence intensity was measured (excitation at 350 nm/emission at 450 nm) for 30 min at 1-min intervals by the microplate reader (BioTek, Winooski, VT). Enzyme activity rate was calculated as ΔOD/min.

### Statistical Analysis

Data are expressed as means ± SE. Each *N* value represents a single fetus. A total number of 20 NMX, 15 early-onset HPX, and 9 late-onset HPX pregnant sows were used to obtain the same number of male and female fetuses, respectively. Fetal heart tissue from the same fetuses were used for quantification of nuclear PGC1α, SIRT1, and SIRT3 expression. Additional animals were used for assays of SIRT3 activity and AMPK because of the limitation of tissue availability for all assays. Fetal body weight characteristics were generated from all of the fetuses used in the study. Statistical analysis was performed using Systat software (San Jose, CA). Comparisons of fetal body and organ weights were made using two-way analysis of variance (ANOVA) using the same NMX group as control. If significant effects were detected (*P*<0.05), post hoc analysis using the Holm-Sidak method was performed to identify differences between groups. Comparisons between protein expression and SIRT3 activity rate were made using Student’s *t* test because only two groups were run on a single gel and comparisons were not made between gels.

## Results

### Effects of HPX on Fetal Body and Heart Weights

Both early- and late-onset hypoxia significantly reduced fetal body weight (*P*=0.008 and *P*=0.006, respectively), and increased relative placental weight (placental wt/body wt ratios) (*P*<0.001 for both) independent of sex compared to age-matched normoxic controls (Table 1). In addition, both early- and late-onset hypoxia significantly increased relative brain weight (*P*<0.001 and *P*=0.04, respectively) similarly between males and females compared to their respective normoxic controls while relative heart weight was unchanged. This is characteristic of asymmetric fetal growth restriction, established in our animal model [13]. Neither food nor water intake rates are affected by intrauterine hypoxia [13], indicating that the fetal growth restriction induced by hypoxia in our animal model is not due to nutrient deficiency, but rather an effect on placental function [30, 31] and/or on fetal growth mechanisms [32].

**Table 1:** Effects of early- and late-onset hypoxia on fetal organ and placental weights

	Males		Females	
	NMX	HPX	NMX	HPX
Early-onset hypoxia				
*N* values	20	15	20	14
*Absolute wt*				
FBW(g)	91.2.0±2.96	81.3±3.73†	87.3±3.16	79.2±3.08†
FHt (g)	0.53±0.02	0.51±0.02	0.50±0.02	0.46±0.02
FBr (g)	2.58±0.03	2.62±0.03	2.55±0.04	2.56±0.04
Plac Wt (g)	4.65±0.24	5.08±0.20†	4.40±0.18	4.94±0.24†
*Relative wt*				
FHt/FBW	0.0057±0.0002	0.0062±0.0002	0.0057±0.0002	0.0059±0.0002
FBr/FBW	0.0287±0.0008	0.0330±0.0014†	0.0298±0.0009	0.0329±0.0012†
Plac/FBW	0.0506±0.0017	0.0633±0.0024†	0.0506±0.0015	0.0623±0.0017†
Late-onset hypoxia				
*N* values	20	9	20	9
*Absolute wt*				
FBW(g)	91.2.0±2.96	80.1±3.60 †	87.3±3.16	77.3±4.06†
FHt (g)	0.53±0.02	0.42±0.02*	0.50±0.02	0.45±0.02*
FBr (g)	2.58±0.03	2.46±0.06†	2.55±0.04	2.44±0.05†
Plac Wt (g)	4.65±0.24	4.97±0.41	4.40±0.18	4.69±0.38
*Relative wt*				
FHt/FBW	0.0057±0.0002	0.0053±0.0002	0.0057±0.0002	0.0058±0.0002
FBr/FBW	0.0287±0.0008	0.0311±0.0015*	0.0298±0.0009	0.0320±0.0011*
Plac/FBW	0.0506±0.0017	0.0618±0.0035†	0.0506±0.0015	0.0602±0.0033†

Comparisons between treatment and sex using 2-way ANOVA with statistical significance (*P*<0.05) indicated by an asterisk differences from their respective NMX control. If *P*<0.05, then a post hoc analysis using Holm-Sidak method was performed to identify differences between groups. There were no sex differences with HPX treatment

*NMX* normoxia, *HPX* hypoxia, *F* fetal, *BW* body wt, *Ht* heart, *Br* brain, *Plac* placenta

*P<0.05

†*P*<0.001

### Expression of Nuclear Protein Expression with Early- and Late-Onset Hypoxia

**Fig. 1:**
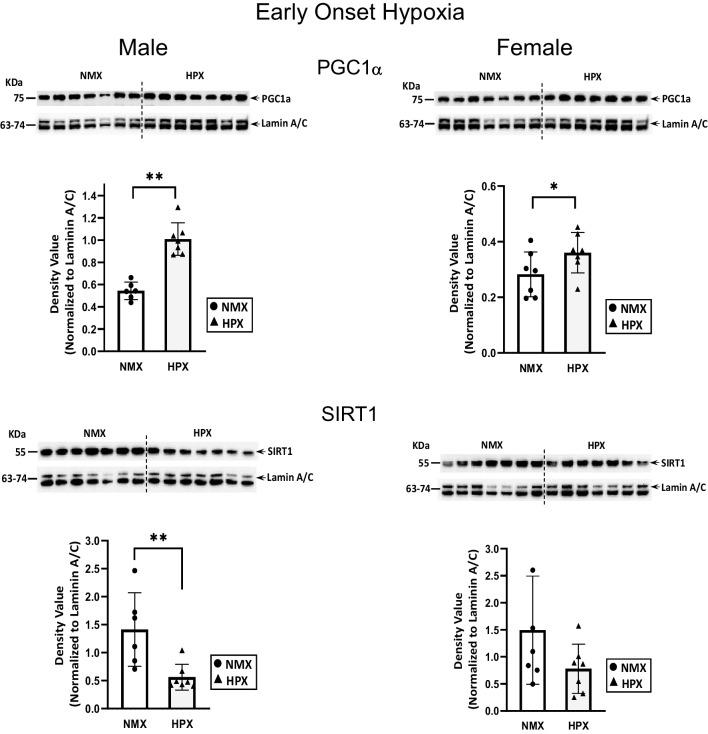
Expression of nuclear PGC1α (peroxisome proliferator-activated receptor γ co-activator 1α) and SIRT1 (sirtuin 1) in normoxic (NMX) and hypoxic (HPX) male (left) and female (right) heart ventricles from fetal guinea pigs whose mothers were exposed to early-onset hypoxia (25-day gestation, 39-day duration, 10.5%O_2_). Density values of target bands (PGC1α or SIRT1) from immunoblots are normalized to Lamin A/C as a loading control. Data are expressed as mean± SEM (*N*=7 for each group). * indicates *P*<0.05 vs NMX, ** indicates *P*<0.01

**Fig. 2:**
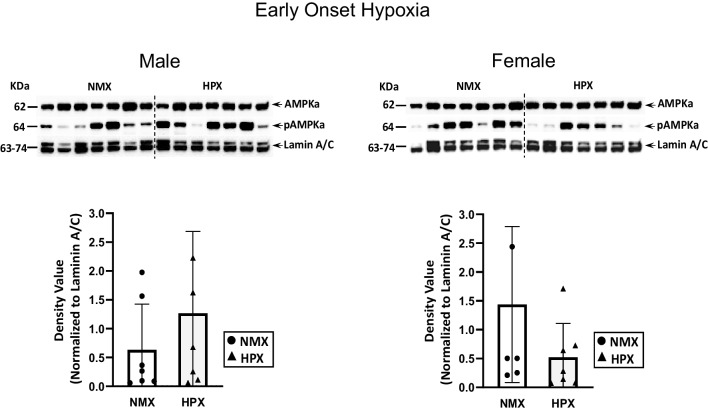
Expression of nuclear pAMPKα (phosphorylated AMP-activated protein kinase) and total AMPKα in normoxic (NMX) and hypoxic (HPX) male (left) and female (right) heart ventricles from fetal guinea pigs whose mothers were exposed to early-onset hypoxia (25-day gestation, 39-day duration, 10.5%O_2_). Density values of target bands (pAMPKα) were normalized to its corresponding total AMPKα for each sample as a pAMPKα/AMPKα ratio. Ratios were normalized to their corresponding LaminA/C loading control and expressed as density values. Data are expressed as mean± SEM (*N*=7 for each group)

Nuclear PGC1α, SIRT1 (Fig. 1), and p-AMPK and total AMPK levels (Fig. 2) were measured in normoxic and hypoxic male and female fetal heart ventricles. Early-onset hypoxia significantly increased nuclear PGC1α in both male (*p*<0.001) and female (*p*=0.04) hearts. Nuclear SIRT1 levels were decreased with early-onset hypoxia in male (*p*=0.004) but not female hearts. Activated nuclear AMPK levels, measured as ratios of p-AMPK/total AMPK, were unaffected by early-onset hypoxia compared to normoxic controls. Although total AMPK levels were highly expressed in the nuclear fractions there was considerable variability in expression of the p-AMPK in both normoxic and hypoxic fetal hearts.

**Fig. 3:**
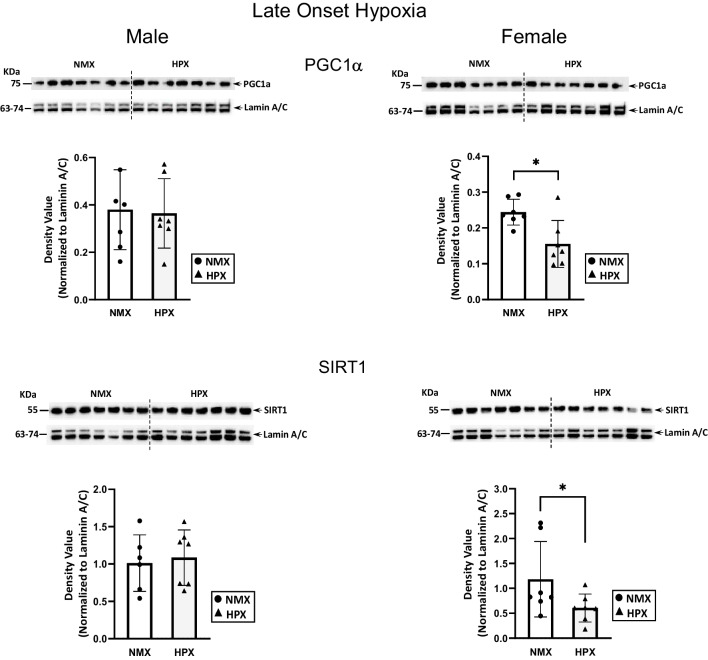
Expression of nuclear PGC1α (peroxisome proliferator-activated receptor γ co-activator 1α) and SIRT1 (sirtuin 1) in normoxic (NMX) and hypoxic (HPX) male (left) and female (right) heart ventricles from fetal guinea pigs whose mothers were exposed to late-onset hypoxia (50-day gestation, 14-day duration, 10.5%O_2_). Density values of target bands (PGC1α or SIRT1) from immunoblots are normalized to Lamin A/C as a loading control. Data are expressed as mean± SEM (*N*=7 for each group), * indicates *P*<0.05 vs NMX

**Fig. 4:**
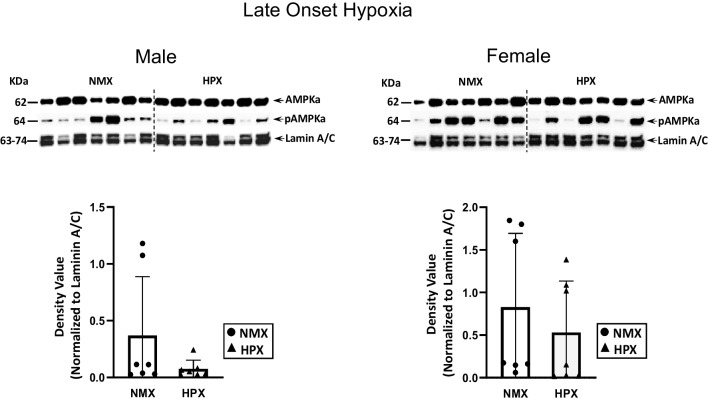
Expression of nuclear pAMPKα (phosphorylated AMP-activated protein kinase) and total AMPKα in normoxic (NMX) and hypoxic (HPX) male (left) and female (right) heart ventricles from fetal guinea pigs whose mothers were exposed to late-onset hypoxia (50-day gestation, 14-day duration, 10.5%O_2_). Density values of target bands (pAMPKα) were normalized to its corresponding total AMPKα for each sample as a pAMPKα/AMPKα ratio. Ratios were normalized to its LaminA/C loading control and expressed as density values. Data are expressed as mean± SEM (*N*=7 for each group)

In contrast, late-onset hypoxia had no effect on nuclear PGC1α levels in male hearts but significantly decreased expression of both PGC1α (*p*=0.004) and SIRT1 (*p*=0.05) in female hearts (Fig. 3). There was no effect of hypoxia on the p-AMPK/total AMPK ratios in either male or female heart tissues (Fig. 4).

### Expression of Mitochondrial Protein Expression with Early- and Late-Onset Hypoxia

**Fig. 5:**
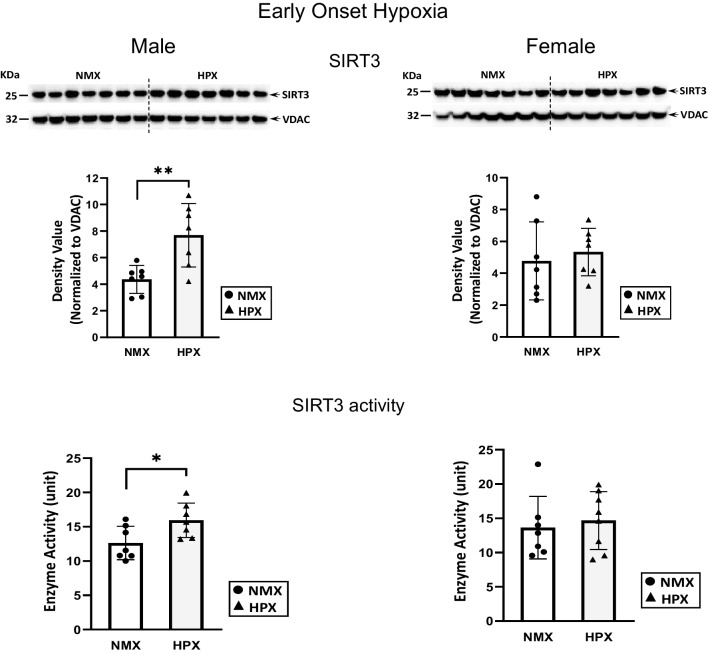
Mitochondrion-associated sirtuin 3 (SIRT3) expression and activity rates in normoxic (NMX) and hypoxic (HPX) male (left) and female (right) heart ventricles from fetal guinea pigs whose mothers were exposed to early-onset hypoxia (25-day gestation, 39-day duration, 10.5%O_2_). Density values of SIRT3 are normalized to VDAC as a loading control. SIRT3 activity is expressed as ΔOD/min units. Data are expressed as mean± SEM (*N*=7 for each group), * indicates *P*<0.05 vs NMX, ** indicates *P*<0.01

**Fig. 6:**
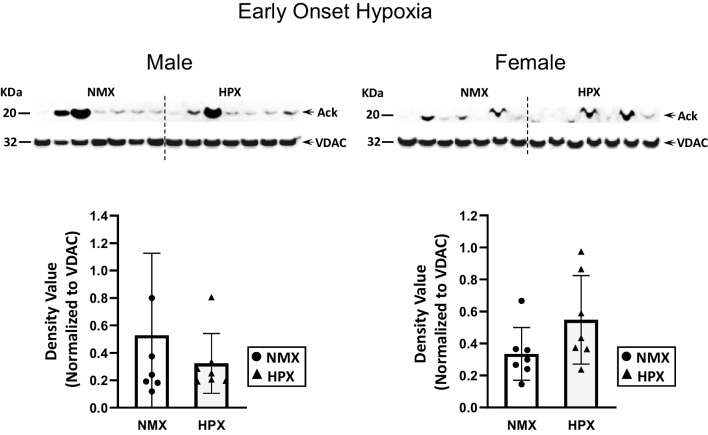
Mitochondrial protein acetylation in normoxic (NMX) and hypoxic (HPX) male (left) and female (right) heart ventricles from fetal guinea pigs whose mothers were exposed to early-onset hypoxia (25-day gestation, 39-day duration, 10.5%O_2_). Density values of target bands (acetylated-lysine) were normalized to VDAC as its loading control. Data are expressed as mean± SEM (*N*=7 for each group)

SIRT3 is a mitochondrion-associated deacetylase important in regulating acetylation of mitochondrial proteins such as complex I and IV subunits, among others [33, 34]. Early-onset hypoxia increased both mitochondrial SIRT3 expression and the activity rate (*p*=0.003 and *p*=0.01, respectively) in male fetal hearts but had no effect in female hearts (Fig. 5). Figure 6 illustrates acetylation of mitochondrial proteins of fetal hearts was identified as a strong band at 20kDa MW similar to that for respiratory complex I, while there was no corresponding band at CIV subunit (not shown) [13]. Immunoprecipitation (inset Fig. 8) confirms that the acetylated band at 20kDa MW corresponds to complex I subunit (NDUFB8). Additional bands were only able to be visualized at longer exposure times but were faint and unable to be quantified. Early-onset hypoxia had no effect on mitochondrial acetylation in either male or female hearts.

**Fig. 7:**
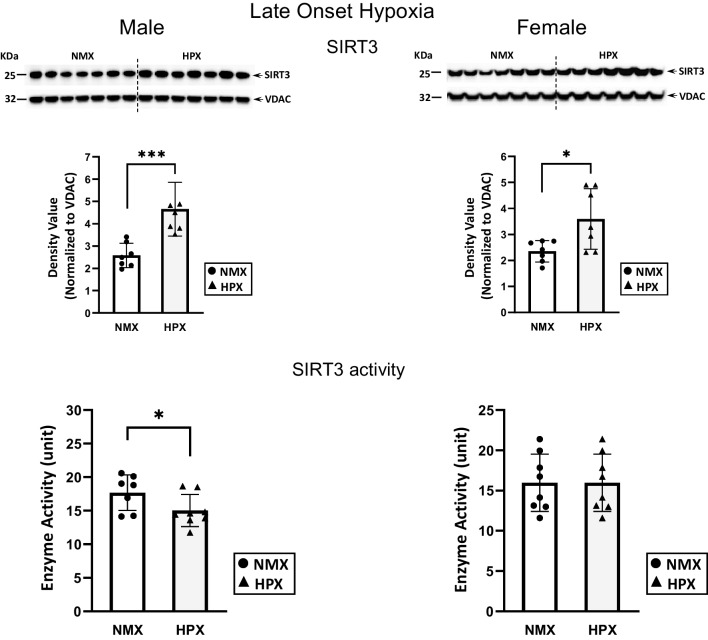
Mitochondrion-associated sirtuin 3 (SIRT3) expression and activity rates of normoxic (NMX) and hypoxic (HPX) male (left) and female (right) heart ventricles from fetal guinea pigs whose mothers were exposed to late-onset hypoxia (25-day gestation, 39-day duration, 10.5%O_2_). Density values of SIRT3 are normalized to VDAC as a loading control. SIRT3 activity is expressed as ΔOD/min units. Data are expressed as mean± SEM (*N*=7-8 for each group), * indicates *P*<0.05 vs NMX, *** indicates *P*<0.005

**Fig. 8:**
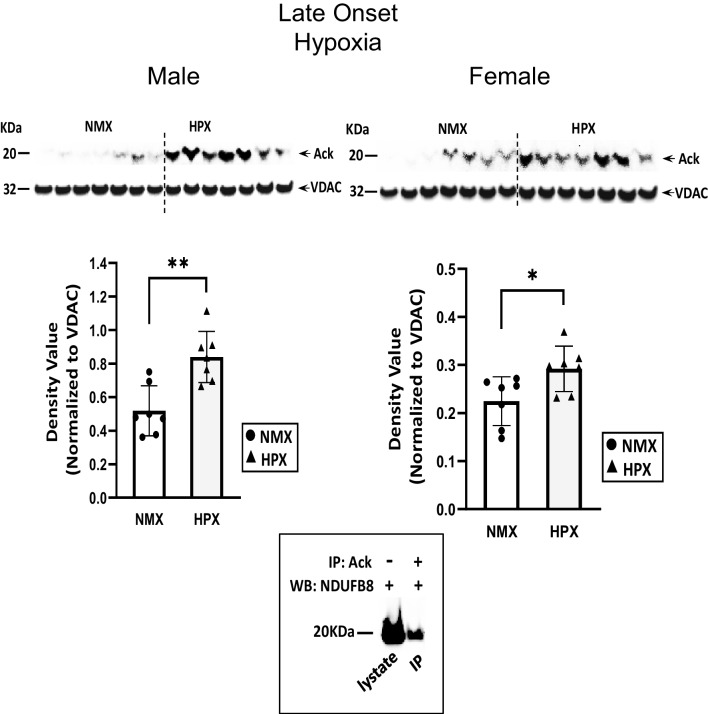
Mitochondrial protein acetylation in normoxic (NMX) and hypoxic (HPX) male (left) and female (right) heart ventricles from fetal guinea pigs whose mothers were exposed to late-onset hypoxia (25-day gestation, 39-day duration, 10.5%O_2_). Density values of target bands (acetylated-lysine) were normalized to VDAC as its loading control. Inset figure shows complex I subunit (NDUFB8) immunocaptured with acetylated-lysine mitochondrial protein at 20kDa MW. Data are expressed as mean± SEM (*N*=7 for each group). * indicates *P*<0.05 vs NMX, ** indicates *P*<0.01

Late-onset hypoxia significantly increased mitochondrial SIRT3 protein expression levels in both male (*p*<0.005) and female (*p*=0.01) hearts (Fig. 7). However, hypoxia inhibited SIRT3 activity (*p*=0.03) in male hearts only. In contrast to early-onset, late-onset hypoxia significantly (*p*<0.05) increased acetylation of mitochondrial proteins in hearts of both males (*p*=0.001) and females (*p*<0.001) (Fig. 8), which corresponded to respiratory complex subunit I (NDUFB8).

## Discussion

Intrauterine hypoxia generates oxidative stress [4, 18, 35, 36], inhibits mitochondrial biogenesis and bioenergetics [37, 38], and disrupts normal fetal heart development [16, 39–42]. Fetal responses to chronic hypoxia include asymmetric fetal growth restriction and placental insufficiency, as well as, redistribution of its cardiac output to critical organs [36], and altered energy metabolism of fetal organs [37].

**Fig. 9:**
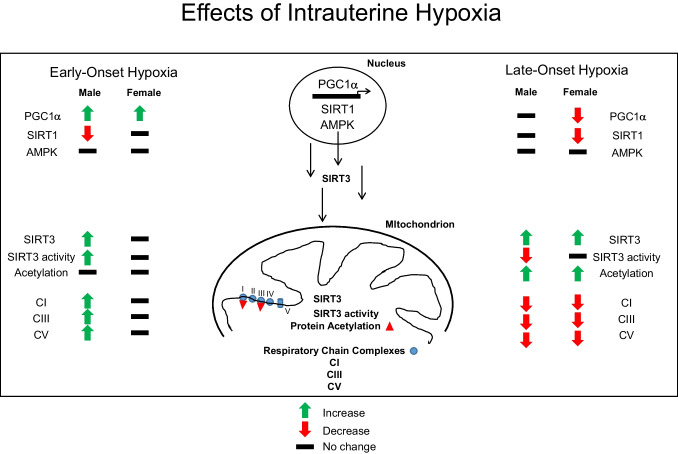
Schematic diagram of effects of intrauterine hypoxia on fetal heart protein expression. The effects of early-onset and late-onset hypoxia on nuclear (PGC1α, SIRT1, and AMPK) and mitochondrial (SIRT3, respiratory chain complex subunits, CI, CIII, CV) protein expression and SIRT3 enzyme activity and mitochondrial acetylation levels of fetal heart ventricle are shown. Arrows (red, decrease; green, increase) and hyphens (no change) indicate the significant directional changes from their respective normoxic controls. Reference to CI, CIII, and CV data is derived from [13]

The first part of the study focused on the effects of hypoxia on nuclear PGC1α, a regulator of mitochondrial biogenesis, and nuclear SIRT1 and AMPK as regulators of PGC1α activity to determine whether chronic hypoxia alters the expression of upstream regulators in a similar direction as downstream targets such as mitochondrial respiratory complex subunits [13]. Effects of hypoxia on PGC1α expression can vary depending on the cell type, redox status of the cell, the presence of inflammation, and severity/duration of hypoxia [43], all of which can occur in hypoxic heart tissue. We previously reported increased expression of respiratory complex subunits (I, III, V) measured with early-onset hypoxia in male fetal hearts and decreased expression of the same subunits with late-onset hypoxia in fetal heart ventricles of both sexes under identical conditions [13] (see Fig. 9). In early-onset hypoxia, the increased PGC1α expression in male fetal hearts corresponded to a compensatory increase in respiratory complex subunits, despite a lack of change in either SIRT1 or activated-AMPK expression. We expected the levels of both of these proteins to be changed in a similar direction as PGC1α. However, nuclear SIRT1 levels were paradoxically decreased with hypoxia and AMPK levels were unchanged. In hypoxic female hearts, early-onset hypoxia increased PGC1α expression but to a lesser degree compared to males, which may account for the lack of change measured in mitochondrial complex subunit expression. In late-onset hypoxia, PGC1α expression was not increased, but either unchanged in male or decreased in female hearts, corresponding to the decreased mitochondrial respiratory complex subunit expression measured in both sexes [13]. In other tissue types, hypoxia increased PCG1α expression in fetal adipose tissue from high altitude pregnant sheep [44], decreased levels in hypoxic cardiomyocyte [45] and adipocyte [46] cell cultures, and decreased expression in chronically (2 and 4 weeks) hypoxic adult mice diaphragm but not skeletal muscle [47]. The current study provides new evidence that intrauterine hypoxia differentially disrupts PGC1α expression in the fetal heart ventricle depending on the timing of gestation and/or duration of hypoxia.

The regulation of downstream mitochondrial biogenesis by PGC1α is complex. Both nuclear- and mitochondrial-encoded proteins are involved in the synthesis, transport, and import into the mitochondria for assembly into respiratory chain complexes [19] via PGC1α signaling. Its expression/activity is regulated within the nucleus [19] by several processes including phosphorylation/acetylation and HIF-1 signaling [48, 49], which increase binding to DNA promoter regions [19] and induce transcription of mitochondrial proteins (i.e., transcription factors NRF1/2, TFAM), as well as, PGC1α itself. A limitation of the study is that a distinct expression pattern of nuclear and mitochondrial protein signaling is difficult to causally link. However, the trends in the overall group of data provide some insight into the significance of the directional changes in expression that occurred with hypoxia. The increased PGC1α signaling induced by early-onset hypoxia may be an adaptive response for preventing the decrease in the mitochondrial complex subunit expression and/or respiratory function in response to hypoxia [13]. In late-onset hypoxia, the lack of increase or decrease in PGC1α signaling is associated with decreased respiratory complex subunit expression, resulting in fetuses more vulnerable to cardiac mitochondrial dysfunction. This has implications regarding postnatal consequences in the programmed offspring. Hearts of prenatally hypoxic (late-onset) guinea pig male offspring exhibit downregulation of mitochondrial respiratory chain complex subunits (CI, CIII, CV) in both ventricular tissue and isolated cardiomyocytes, as well as, decreased respiratory function (i.e., maximal oxygen consumption, respiratory reserve capacity), compared to their normoxic age-matched controls [12]. In the same study [12], prenatal hypoxia reduces contractile function (e.g., stroke volume, cardiac output, ejection fraction, fractional shortening) measured by ultrasound of offspring hearts [12]. Both studies, combined, suggest an important programming effect of intrauterine hypoxia on disruption of PGC1α signaling and mitochondrial protein expression in fetal hearts with consequences manifest in the offspring. Yet, prenatally hypoxic female offspring hearts did not exhibit sustained effects on either contractile or mitochondrial function compared to males, despite hypoxic effects on PGC1α signaling and mitochondrial protein expression in female fetal hearts. Thus, there may be secondary environmental responses, postnatally, that are adaptive in female offspring.

The second part of the study investigated the effects of hypoxia on mitochondrial SIRT3 expression/activity and protein acetylation. Mitochondrial acetylation reduces mitochondrial function via inhibiting ATP generation, hormone synthesis, and calcium regulation [50]. SIRT3 is a mitochondrial deacetylase encoded in the nucleus, transported to and imported into the mitochondria where it regulates acetylation of mitochondrial proteins [51, 52] such as complexes I, II, and IV [31, 32, 53]. With late-onset hypoxia, the increased mitochondrial acetylation corresponds to the decrease in SIRT3 activity in males or no change in females. The compensatory increase in SIRT3 expression may limit the effects of SIRT3 activity although not sufficient enough to prevent the increased acetylation. An alternative may be that other mitochondrial SIRTs (i.e., SIRT4, SIRT5) [54] whose activities may be decreased by hypoxia contribute to the increased acetylation levels. Interestingly, the increased acetylation is associated with respiratory complex I subunit (NDUFB8), which SIRT3 has been reported to deacetylate [55]. In contrast, early-onset hypoxia did not increase mitochondrial acetylation in either sex. In males, there was a greater increase in SIRT3 expression compared to late-onset hypoxia along with increased activity, which may contribute to a protective response in preventing increased acetylation. Overall, increased mitochondrial acetylation, which targets complex I, imposes a great risk to mitochondrial respiration in hypoxic fetal hearts due to its potential inhibitory actions on the respiratory chain [31, 55].

Sex differences in fetal cardiac protein expression occurred in response to chronic hypoxia with regard to PGC1α and SIRT3, along with SIRT3 activity. The combined increase in levels of PGC1α, SIRT3 expression/activity, and mitochondrial respiratory complex subunits in early-onset hypoxic male but not female hearts identifies a compensatory capacity that is somewhat sex-related. With late-onset hypoxia, however, PGC1α was decreased in females but not males although both sexes increased SIRT3 expression, mitochondrial acetylation, and decreased respiratory complex subunit expression [13]. The mechanisms underlying sex differences between fetuses and adults differ, with the former relying on genetic and/or epigenetic influences [56, 57]. Mitochondria of adult female hearts exhibit a greater functional advantage over mitochondria from males because of a greater antioxidant capacity, lower generation of reactive oxygen species, greater energy production, and lower calcium uptake [56, 58]. This is attributed, in part, to a hormonal effect of estrogen on mitochondrial function and biogenesis, regulated by binding to both ERα in the nucleus and ERβ in the mitochondrion [59, 60]. While we did not evaluate baseline sex differences, sex-related responses to hypoxia appear compensatory with early-onset and decompensatory with late-onset hypoxia, regarding PGC1α signaling, SIRT1 expression/activity and respiratory complex subunit expression.

Overall, exposure to hypoxia in early gestation initiates adaptive mechanisms that protect the fetal heart with regard to mitochondrial respiration prior to birth or has a lesser impact due to reduced mitochondrial maturity [6, 61]. The cardiac response to hypoxia in late gestation, however, shows a decompensation with no change or decreased PCC1α signaling, decreased mitochondrial respiratory subunit expression [13], and increased mitochondrial acetylation. The reduced capacity of fetal hearts to adaptively respond to hypoxia in late gestation may be due to a shorter time interval and/or a reduced plasticity of cellular mechanisms for compensation. This identifies a differential capacity of the fetal heart mitochondria to respond to chronic hypoxia depending on the conditions, which may increase the risk of mitochondrial dysfunction, contributing to cardiovascular programming of the offspring [15, 57].
